# PSYCHOMETRIC PROPERTIES OF THE NORWEGIAN VERSION OF THE PEDIATRIC FAMILY NEEDS QUESTIONNAIRE – PEDIATRIC (FNQ-P)

**DOI:** 10.2340/jrm.v58.45390

**Published:** 2026-10-07

**Authors:** Edel J. SVENDSEN, Marianne LØVSTAD, Ingvil LABERG HOLTHE, Ida Marie BORGEN, Cathrine BRUNBORG, Nina ROHRER-BAUMGARTNER

**Affiliations:** 1 Center for Research and Education, Sunnaas Rehabilitation Hospital, Nesodden; 2 Department of Nursing and Health Promotion, Oslo Metropolitan University, Oslo; 3 Department of Psychology, Faculty of Social Sciences, University of Oslo, Oslo; 4 Department of Physical Medicine and Rehabilitation, Oslo University Hospital, Oslo; 5 Oslo Centre for Biostatistics and Epidemiology, Oslo University Hospital, Oslo, Norway

**Keywords:** family needs, acquired brain injury, children, paediatrics, reliability

## Abstract

**Objective:**

Evaluate test–retest reliability, internal consistency, and construct validity of the Norwegian version of the Pediatric Family Needs Questionnaire (FNQ-P).

**Design:**

Test–retest reliability, internal consistency, and construct validity.

**Subject/patients:**

Parents/caregivers of children (6–17 years) with ABI in the chronic phase who participated in the Child in Context Intervention (CICI) study.

**Methods:**

Internal consistency was assessed with Cronbach’s alpha, while test–retest reliability was explored with intraclass correlation coefficients (ICC), *t*-tests, and coefficients of variation. Standard error of measurement and minimal detectable change were also assessed. Construct validity in relation to Matrices and Similarities from WISC-V, Health and Behavior Inventory (HBI), Behavior Rating Inventory of Executive Function-2 (BRIEF-2), Pediatric Quality of Life Inventory (PedsQL), Tool to Measure Parenting Self-Efficacy (TOPSE), Patient Health Questionnaire-9 (PHQ-9), and Generalized Anxiety Disorder Scale-7(GAD-7) was explored with correlation coefficients.

**Results:**

Test–retest reliability of the total mean FNQ-P score was good (ICC = 0.84). Internal consistency was excellent with a Cronbach’s alpha of 0.98. Construct validity was confirmed. The only significant correlations were weak correlations with parent anxiety (GAD-7) and child’s emotional functioning (PedsQL).

**Conclusion:**

The Norwegian FNQ-P has good psychometric properties and can be used to obtain reliable and valid data regarding family needs.

## LAY ABSTRACT

Families with a child that has suffered a brain injury may have emotional, cognitive, behavioural, and social problems that last for a long time, often years, after the injury. Not all families receive the help they need after brain injury, and there has been a lack of good measures to assess families’ perceived healthcare needs. In this paper we show that a Norwegian version of the questionnaire, “The Pediatric Family Needs Questionnaire (FNQ-P)”, seems to perform well as a measure of healthcare needs and that results are stable over time. With these data in place, we recommend that the FNP-Q is used in the Norwegian clinical and scientific setting to provide information in a standardized way, and which can be communicated internationally.

Children with paediatric acquired brain injury (pABI) often experience persistent cognitive, emotional, social, and behavioural difficulties. Healthcare needs of families with children with pABI are often unmet (1, 2), resulting in negative long-term consequences on family functioning and relationships (3). Healthcare needs may encompass both the family’s need for information concerning the child’s health, as well as various forms of support, including emotional, instrumental, professional, and community assistance. Instrumental support may refer to practical help to meet the physical needs of the child, and family members. Community support may refer to support within the child’s environment, such as ensuring that the school understands the child’s challenges and symptoms. Despite the potential negative consequences of unmet healthcare needs after pABI, available methods for needs assessment vary greatly and typically lack adequate psychometric properties (4, 5).

Ongoing care needs and service gaps related to both short- and long-term support have been identified in families with pABI, in addition to social support needs and needs related to return to school (6, 7). Healthcare for children with pABI is often insufficiently coordinated or integrated with educational systems, and the need for rehabilitation and cognitive/educational services has been shown to be higher than the actual services provided (5). Further gaps are related to unmet needs related to child behaviour management and caregiver stress and coping (8). In a review of the needs of affected children and family members after pABI (9), person-related needs were adolescent-specific needs, need for support with cognitive, social, emotional, and physical problems, and assistance with practical challenges. Service-related needs, such as medical information, medical therapies, and educational needs, were consistently high after discharge from hospital for children with moderate to severe traumatic brain injury (TBI). In addition, families’ need for psychological, caregiver, and family support services increased in the months post-discharge, as did the probability of unmet needs (9). Unmet needs were particularly related to information, emotional support, and care transitions, which were intensified by healthcare providers’ limited understanding of the children’s difficulties. Time since injury has been associated with a higher likelihood of unmet needs during transitions (4), and caregivers of children with preexisting psychosocial conditions are more likely to report unmet needs (3). Furthermore, parental perception of unmet healthcare needs is strongly related to family burden (10).

In summary, it is important to monitor unmet needs after pABI, because the injury may have severe impact on the family if needs are not recognized and met. However, most of the literature on needs for this patient group is based on variants of semi-structured and unstandardized interviews with caregivers, or on professionals’ needs assessment. Hence, there is a need for psychometrically sound and validated tools that can assess a broad range of needs as reported by the families themselves.

The Family Needs Questionnaire Revised (FNQ-R) is a well-validated tool designed to measure to what extent families’ needs have been met in adults with ABI (11). To capture family needs after pABI, a paediatric version (FNQ-P) has been developed (12). The FNQ-P assesses family members’ perceptions of whether their needs have been met at a given timepoint and measures changing needs over time. The FNQ-P consists of 40 items and provides a total score and the degree to which the needs of families have been met across 6 categories: Health Information, Emotional Support, Instrumental/Practical Support, Community Support, Professional Support, and Involvement with Care. The original English version of the FNQ-P and translations into Swedish and Lithuanian have previously been documented to have good test–retest reliability, internal consistency, and construct validity (13, 14).

The FNQ-P has been translated into Norwegian by an expert group following a 10-step procedure and using forward–backward translation with a professional translator (15). Some minor cultural adaptations were performed, and cognitive interviews conducted. However, the psychometric properties of the Norwegian version of FNQ-P have not yet been established.

The aim of the current study is to explore the psychometric properties of the Norwegian version of the FNQ-P. The specific aims are to:

1. evaluate the test–retest reliability of two FNQ-P administrations,
2. assess the internal consistency of FNQ-P, and
3. assess construct validity by evaluating the correlation between the FNQ-P and related constructs (overall family functioning, child symptom level, and parent self-efficacy and psychiatric symptoms).

## METHODS

### Sample

The study sample was derived from a randomized controlled trial (RCT) investigating the effect of a goal-oriented intervention in the chronic phase of paediatric acquired brain injury: the Child in Context Intervention (CICI) (16). Inclusion criteria were school-aged children (6–16 years) with clinical ABI diagnosis and CT/MRI-verified insult-related intracranial abnormalities or loss of consciousness and verified neurological symptoms post-injury in cases where MRI could not be administered. Minimum time since injury was 1 year. The children were required to attend school regularly, and the family needed to be able to actively participate in goal-oriented work, including having internet access and speaking sufficient Norwegian. An exception could be made for parents who spoke English but were able to understand and read study-related information in Norwegian. Children with severe pre- or comorbid neurological or neuropsychiatric disorders that would confound assessment and/or treatment outcomes, children with brain tumours or cancer in active treatment or at great risk of relapse, and children with severe psychiatric illness or injuries so severe that they were in institutionalized care most of the time were excluded, along with families with severe parental mental health disorders, drug abuse, or indications of a history of or risk of domestic violence (16).

### Measures

The FNQ-P (12) is scored on a 5 point Likert scale from 1 to 5 whether responses correspond to 1 = “not at all met”, 2 = “met very little”, 3 = “somewhat met”, 4 = “met a lot”, or 5 = “completely met”, i.e., higher scores indicating a higher degree of met needs. Each item may also be scored as “not needed” if there is no need at the time of administration. The current study investigated reliability and validity of the total mean score, category scores, and individual item scores. The original English version of the FNQ-P has showed good test–retest reliability, with an intraclass correlation coefficient of 0.84. With regard to internal consistency, the Cronbach’s alpha for the FNQ-P total scale was excellent at 0.94 (0.83–0.93) (14).

### Measures used in assessing construct validity

The relationship between the FNQ-P total mean score and other parent-reported measures related to the parents (family functioning, parenting self-efficacy, and parental anxiety and depression) was explored. We also examined the relationship of the FNQ-P with the children’s level of executive functioning in daily life, abstract reasoning abilities, brain injury symptom levels, and quality of life.

Family functioning was assessed with parent reports on the 12-item, General Functioning subscale of the McMaster Family Assessment Device (17), which has high internal consistency (18). High scores indicate worse family functioning and a scaled (mean) score of 2 or more is regarded as an indication of problematic family functioning. The FAD-GF total score was applied.

Parenting self-efficacy was measured with the Tool to measure Parenting Self-Efficacy (TOPSE) (19), which exists in a child and an adolescent version. The latter was used for parents of children of 10 years of age and older. The TOPSE is rated on a scale from 0 (completely disagree) to 10 (completely agree). Higher mean total scores indicate higher parenting self-efficacy. Reliability has been shown to be excellent. Mean total and mean subscale scores were applied.

Symptoms of depression and generalized anxiety in parents were assessed with the Patient Health Questionnaire-9 (PHQ-9) (20) and the Generalized Anxiety Disorder Scale-7 (GAD-7) (21) respectively. Both have excellent internal consistency. Respondents reported the frequency of symptoms on a scale from 0 to 3. In both questionnaires, negatively worded questions are reversed, yielding scores from 1 to 4. Total scores of 0–4 are considered as “minimal”, 5–9 as “mild”, 10–14 as “moderate”, 15–19 as “moderately severe”, and 20–27 as “severe” symptom levels.

Brain injury symptoms were reported by parents on the Health and Behavior Inventory (HBI) (22), which consists of 20 items that measure cognitive (11 items) and somatic symptoms (9 items). Symptoms are scaled using a 4-point Likert scale ranging from 0 “never” to 3 “often” (range cognitive subscale 0–33 and somatic subscale 0–27), with higher scores indicating more symptoms. Cronbach’s alpha has been shown to be good to excellent a few months after injury (23). Mean HBI total and mean subscale scores were used.

Non-verbal and verbal abstract reasoning abilities in the children was measured with the scaled scores of the Matrices and Similarities subtests from WISC-V (24). The 63-item parent version of the BRIEF-2 (25, 26) was used to assess executive dysfunction in daily life. Items are scored as 0 “never”, 1 “sometimes”, and 2 “often”, with higher scores representing worse functioning. The Global Executive Composite (GEC), which is a summary score of all 10 subscales, is used here. Cronbach’s alpha for composite scores of the BRIEF-2 has been shown to be good to excellent in clinical and nonclinical populations.

Children’s quality of life was assessed with the 23-item core module of the Peds-QL (27), which has demonstrated excellent reliability and validity. Items are scored from 0 to 4 and signify the following: 0 (never a problem), 1 (almost never a problem), 2 (sometimes a problem), 3 (often a problem), and 4 (almost always a problem). Higher scores indicate higher quality of life. Parent-reported total mean and subscale scores were applied.

### Data sampling methods and study design

This study was conducted at Sunnaas Rehabilitation Hospital (SRH). Questionnaire measures were answered by the parents in an electronic data capture system, Viedoc™ (Viedoc Technologies AB, Uppsala, Sweden). Data were collected between May 2021 and December 2022.

The first measuring point in the current study (T1) coincides with the baseline assessment in the RCT. Sociodemographic and injury-related data were recorded at T1 through clinical records and a semi-structured interview with parents. Participants were asked to answer the FNQ-P a second time after 1 week (T2). Both measurement points were before randomization in the clinical trial. Both parents and/or legal guardians were asked to respond to the FNQ-P individually, providing more than 1 possible sampling point per child.

### Ethics

The study was approved by the Norwegian Regional Committee for Medical and Health Research Ethics (2019/1283) and the Data Protection Officer at SRH and complies with the Helsinki Declaration. Sixteen-year-olds and their parents provided written informed consent. Children under the age of 16 received age-appropriate information regarding the study and assented to participate. In line with Norwegian legislation and ethical requirements parents consented on their behalf.

### Statistics

Sample characteristics are provided with descriptive statistics. FNQ-P test–retest reliability was explored with intraclass correlation coefficients (ICCs; two-way mixed, random absolute agreement, single measures) with a target of an ICC > 0.80 (indicative of excellent reliability) (28, 29) and targeting a lower limit of the 95% confidence interval (CI) of > 0.50 (at least moderate reliability) (29). ICCs were calculated for total mean, subscale means, and single items of the FNQ-P. A paired sample *t*-test was conducted to evaluate differences between test and retest scores in the overall sample, and effect size (Cohen’s *d*) was calculated with a target of a small effect size (i.e., 0.20 *d* < 0.50) (30, 31). Coefficient of variation (CoV) was applied as a parallel measure of reliability for the entire sample, using calculation of method error


$$\left(ME\right)=\frac{SD_{\mathrm{diff}}}{\sqrt{2}}$$

and CVME = 2ME/(X1+ X2)×100, where X1 and X2 are the mean group scores for test and retest. A target CoV of < 10% was set as indicative of excellent reliability (31). Internal consistency was explored with Cronbach’s alpha.

As the FNQ-P can be used as an outcome measure in clinical trials, standard error of measurement (SEM) was computed where SEM = SD*√(1-ICC) and minimal detectable change (MDC80) was then calculated at 80% CI where MDC80 = SEM*1.28*√2 (30). The percentage MDC compared with overall FNQ-P total score/100 was then computed – this relative quantity permits comparison of the magnitude of measurement error across measures. An MDC percentage estimate of less than 30% has been recommended as being indicative of acceptable measurement error (32). To further investigate agreement, a Bland–Altman plot was used to assess the mean difference and limits of agreement (LoA) between test and retest scores in the overall sample (33, 34). The upper and lower LoA are defined as the mean difference between the measuring methods ± 1.96 SD of the mean difference.

Construct validity was explored with correlation coefficients. As most FNQ-P scales were non-normally distributed, Spearman’s correlation coefficients were applied. We adopted Altman’s guidelines, in which *r* values below 0.20 are considered poor, 0.21 to 0.40 fair, 0.41 to 0.60 moderate, 0.61 to 0.80 good, and above 0.81 very good (34).

## RESULTS

### Sample characteristics, including FNQ scores

Table I describes characteristics of the subsamples included. The total T1 RCT sample includes baseline data from all families in the CICI study, which is the data that Cronbach’s alpha, correlations, and regressions are based on. The T2 retest sample represents data from the 46 families where 1 or both parents responded to FNQ-P at the T2 reliability time point. Mean time between the 2 assessment time points was 10.36 days (SD 4.36), range 7–20.

**Table I:** Sample characteristics

Item	Total T1 RCT sample (*n* = 111)	T2 retest sample (*n* = 77)
Child’s age at T1, Median (IQR); range	11.67 (6.00); 6.25–16.92	11.67 (5.64); 6.25–16.92
Child’s sex: *n* (%)		
Boys	30 (51.7)	25 (54.3)
Girls	28 (48.3)	21 (45.7)
Injury aetiology *n* (%)		
TBI	16 (27.6)	15 (32.6)
Stroke	10 (17.2)	8 (17.4)
Anoxia	6 (10.3)	4 (8.7)
Tumour	15 (25.9)	12 (26.1)
Infection	8 (13.8)	6 (13)
Other	3 (5.2)	1 (2.2)
Age at injury, mean (SD); range	6.3 (7.48); 0–14.50	6.63 (8.27): 0–14.5
Time since injury (years)	6.00 (2.39); 1.25–16.92	5.25 (5.60); 1.25–16.92
Relationship to child, *n* (%)		
Mother	59 (53.2)	43 (55)
Father	52 (46.8)	34 (45)

### Test–retest reliability

Test–retest reliability of the total mean FNQ-P score was good (ICC = 0.84). Category ICCs varied from moderate to good (range 0.62–0.80). Lower 95% CI bounds were above the preset limit of 0.5 for all categories except Professional support, which was 0.47. Single-item ICCs varied from 0.46–0.85, with only 2 being marginally below 0.5 (Item 40: 0.46 and Item 20: 0.47) (see Table I for details). An SEM of 0.22 was demonstrated, along with an MDC of 0.4 points, and MDC% of 12.6, which is well below the targeted maximum of 30%. The CoV for the total mean FNQ-P was 11.6 %, i.e., slightly below the predefined excellent level.

Only 1 item (#32 – To have my wishes respected if I don’t want help) was rated as not needed by at least 25% of respondents at baseline, and none at T2, where this item was rated as not needed by 21%. In no cases did more than 20% of single items differ by more than 2 points from T1 to T2 (Table II).

**Table II:** Test–retest and internal consistency of FNQ-P total mean, categories, and individual items

Item number	FNQ-P total mean score (SD)		ICC	95% CI of ICC	Test-retest scores different by > 2 points: # of participants	Number of “not needed” response used at T1 ^Φ^
	T1 RCT sample (*n* = 112)	T2 reliability sample (*n* = 88)				
**Total mean score: Cronbach’s alpha 0.98**	2.53 (0.75)	2.6 (0.73)	0.84	0.76; 0.89		
**Health Information: Cronbach’s alpha 0.93**	2.74 (0.80)	2.73 (0.82)	0.77	0.66; 0.85		
Changes in health status	3.16 (1.07)	3.08 (0.93)	0.62	0.43; 0.75	3	16
Abilities in future	2.48 (0.89)	2.34 (0.93)	0.62	0.46; 0.74	5	3
Care needs	2.7 (0.93)	2.88 (1.00)	0.52	0.32; 0.67	6	5
Understandable information	3.17 (0.99)	3.18 (1.01)	0.62	0.46; 0.75	3	8
Information on rehab	2.61 (1.02)	2.62 (1.16)	0.66	0.46; 0.80	3	33
Questions answered thoroughly/respectfully	3.18 (1.01)	3.14 (0.97)	0.62	0.45; 0.75	3	14
Care management brain injury	2.58 (1.01)	2.6 (1.06)	0.67	0.50; 0.79	3	20
Information on physical challenges	2.81 (1.05)	2.65 (1.02)	0.55	0.34; 0.71	5	16
Information on thinking challenges	2.66 (0.92)	2.51 (0.89)	0.58	0.40; 0.72	5	10
Impact of drugs and alcohol	1.41 (0.87)	1.52 (0.89)	0.65	0.44; 0.79	3	33
**Emotional Support: Cronbach’s alpha 0.92**	1.96 (0.84)	2.15 (0.86)	0.80	0.70; 0.87		
Share feelings with other parents	1.81 (0.98)	2 (0.99)	0.85	0.76; 0.91	0	16
Access to counselling	1.81 (0.99)	2.24 (1.16)	0.63	0.44; 0.77	7	18
Help accepting child’s changes	1.96 (0.95)	1.97 (0.88)	0.58	0.38; 0.73	3	14
Support for hope	2.0 (1.02)	2.16 (1.04)	0.75	0.60; 0.84	2	16
Normalize mixed feelings	2.04 (1.04)	2.13 (1.06)	0.63	0.44; 0.77	2	23
Help remaining realistic	1.97 (0.93)	2.10 (0.95)	0.79	0.67; 0.87	1	15
**Instrumental Support: Cronbach’s alpha 0.79**	2.11(0.94)	2.04 (0.94)	0.66	0.50; 0.78		
Help educating family + friends	1.85 (0.93)	1.82 (0.93)	0.61	0.41; 0.76	3	27
Help with other children’s needs	1.99 (1.07)	2.11 (1.1)	0.76	0.63; 0.85	3	24
Support for parent’s needs	1.82 (1.02)	1.88 (1.01)	0.47	0.24; 0.64	5	23
Practical support in rehab	2.41 (1.13)	2.48 (1.11)	0.55	0.33; 0.72	7	25
**Involvement with Care: Cronbach’s alpha 0.92**	3.07 (0.96)	3.17 (0.83)	0.8	0.68; 0.87		
Help with child’s transitions	2.94 (1.11)	3.06 (0.97)	0.65	0.46; 0.79	3	22
Communication of care plan	2.86 (1.10)	2.83 (1.05)	0.71	0.55; 0.82	1	19
Wishes respected	3.43 (1.41)	3.6 (1.16)	0.80	0.6; 0.9	1	63^Φ^
Respect from professionals	3.41 (1.08)	3.56 (1.08)	0.66	0.49; 0.79	5	14
Opinions and wishes listened to	3.26 (1.02)	3.31 (1.01)	0.68	0.50; 0.81	3	21
Involved in care	2.86 (1.17)	2.81 (0.99)	0.51	0.27; 0.69	7	21
Access to and information on chart content	2.96 (1.25)	3.05 (1.17)	0.7	0.53; 0.81	6	17
Included in treatment programme	3.01 (1.13)	3.02 (1.03)	0.67	0.50; 0.80	3	22
**Professional Support: Cronbach’s alpha 0.88**	2.41 (0.85)	2.57 (0.82)	0.62	0.47; 0.74		
Collaboration with school/rehab team	2.9 (0.92)	3.07 (1.00)	0.52	0.32; 0.67	6	5
Understand injury impact in family	2.04 (1.02)	2.09 (0.93)	0.63	0.62; 0.86	4	15
Help advocate academic support	2.6 (1.15)	2.79 (1.01)	0.6	0.43; 0.74	9	9
Help decide child’s independence	2.2 (1.04)	2.35 (0.85)	0.52	0.31; 0.68	7	16
Help describe child’s strengths	2.48 (1.04)	2.67 (0.93)	0.68	0.50; 0.81	2	24
Help manage emotions/behaviour	2.01 (0.96)	2.25 (1.14)	0.51	0.29; 0.68	10	12
**Community Support: Cronbach’s alpha 0.81**	2.44 (2.00)	2.51 (0.81)	0.73	0.60; 0.82		
Access to contact info for help	2.59 (1.31)	2.58 (1.29)	0.73	0.60; 0.83	6	8
Info on accessing future support	1.69 (0.92)	1.74 (0.87)	0.72	0.57; 0.82	2	13
Sharing child’s skills with community	2.83 (0.93)	3.01 (0.93)	0.67	0.52; 0.78	5	4
Information about education	2.65 (1.05)	2.71 (0.95)	0.61	0.42; 0.74	5	8
Resources for child	2.11 (0.99)	2.29 (0.82)	0.62	0.43; 0.76	2	19
Resources for family	2.64 (1.16)	2.68 (1.02)	0.46	0.23; 0.64	5	21

ICC: intraclass correlation coefficient; CI: confidence interval.

Φ Item where > 25% of respondents used N/A option.

With regard to internal consistency, the Cronbach’s alpha for the FNQ-P total mean scale at T1 was excellent at 0.98, varying between 0.79 and 0.93 among categories. See Table II for details.

In the Bland–Altman plot (Fig. 1), there was no systematic pattern on the y-axis, which suggests that there is no indication of proportional bias at any point in the range. This suggests that the difference between the 2 administrations of the FNQ-P does not change with the measurement score. Also, the average difference in mean scores between the FNQ-P administered at T1 and at the retest time point is close to 0, indicating very little systematic bias. Moreover, the LoA range was narrow (less than 1 point in each direction) and there were few outliers, indicating good agreement. The paired sample *t*-test involving the Total mean score at the 2 measuring points did not indicate significant difference (*p* = 0.34), and the effect size was small (*d* = 0.42).

**Fig. 1:**
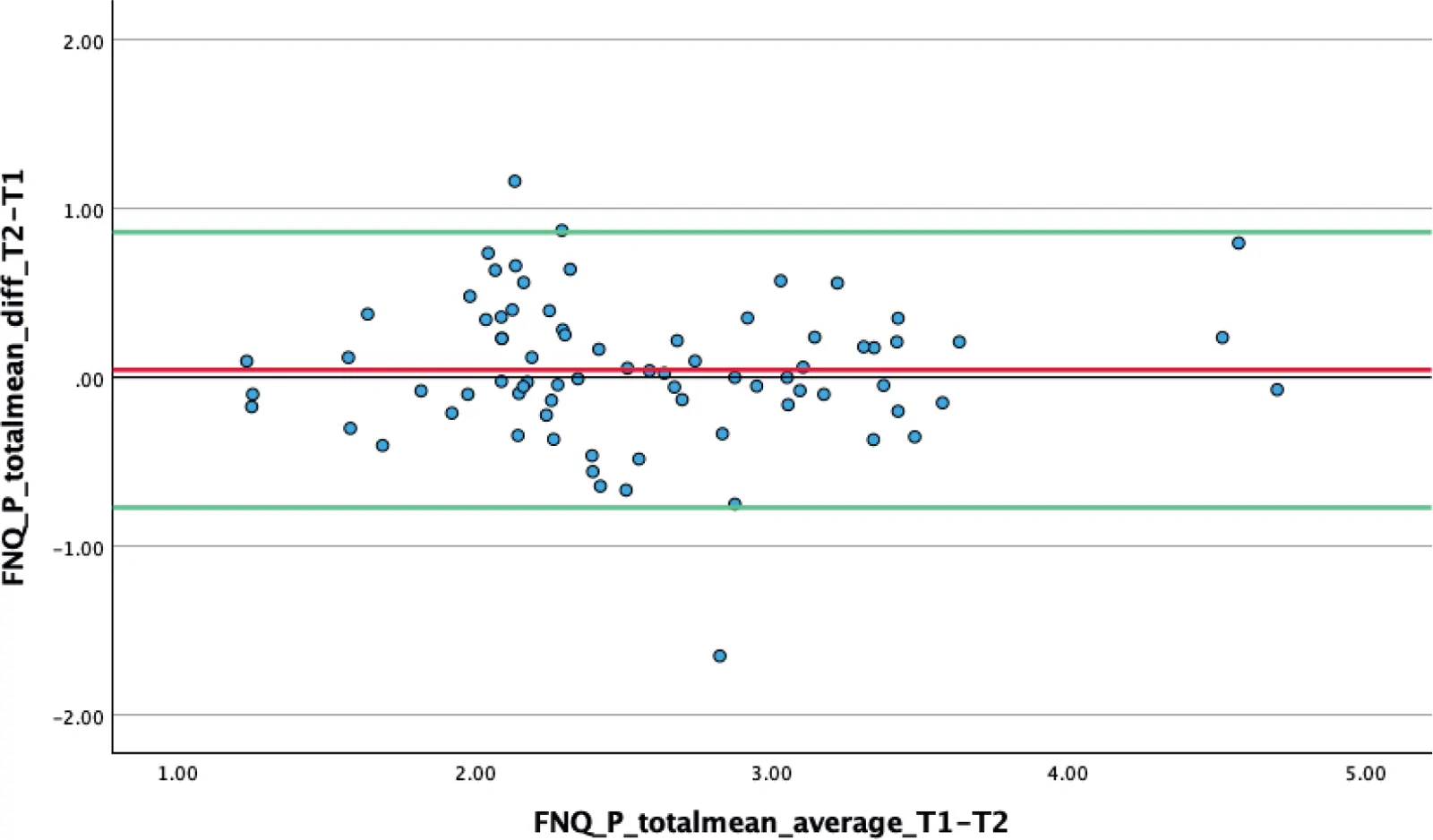
Bland–Altman plot for the total reliability sample (*n* = 77). The horizontal lines represent the mean (red line) and ± 1.96 SD (green lines) of the difference between test (T1) and retest (T2) scores.

### Construct validity

The only significant correlations between FNQ-P total mean scores and variables in the validity exploration were a weak negative and significant correlation with parental anxiety levels on the GAD-7 (*r* = –0.19, *p* = 0.047), and a fair correlation with the PedsQL emotional functioning subscale (parent: *r* = 0.271, *p* = 0.004; child: *r* = 0.302, *p* = 0.042).

## DISCUSSION

This study examined the test–retest reliability, internal consistency, and construct validity of the Norwegian version of the paediatric FNQ-P in a sample that participated in an individualized and goal-oriented rehabilitation trial in the chronic phase of paediatric acquired brain injury. The results overall demonstrated excellent psychometric properties with stable results over the retest period, and excellent internal consistency for the total scale. Indicators of measurement variance suggest that the Norwegian FNQ-P is well suited to be used as an outcome measure in clinical trials. With regard to convergent validity, results indicate that the FNQ-P measures a distinct construct, and that unmet needs are weakly associated with emotional functioning in both parents and children.

### Time between measurements

Time between measurements was 10.36 days (SD 4.36), with a range of 7–20, which is within the suggested interval of 2 to 14 days, with a range up to 21 days (35), and within the average for test–retest reliability studies in patient-reported outcome (36). Of note, Gan et al. (13) had a mean 3-week test–retest window for the English version, which might explain the higher ICCs in the current study.

### Reliability and validity

There are so far few published papers on the FNQ-P, making comparisons with other studies difficult. However, Gan et al. (13) investigated validity of the English, Swedish, and Lithuanian versions. They found a total ICC at the 0.8 level, with the lowest ICCs for the Lithuanian version, i.e., 0.74, which was also the sample with more newly injured children. However, the samples were small when split by language. Overall, the Norwegian FNQ-P demonstrated good test–retest reliability, with ICC for the total score comparable to the original version, and with the lower bounds of the 95% confidence intervals exceeding the preset limit of 0.5 for most categories. However, the professional support category had a lower bound marginally below this threshold, i.e., 0.47. This may be because this category comprises items ranging from professional help at school to health/rehabilitation, as well as emotional support and items connected to the child’s independence. Further investigation and potential refinement of the items in this category is recommended. The internal consistency of the Norwegian version was also excellent and comparable to that of the original FNQ-P. The only single item that was scored as not relevant by more than 25% of respondents at T1 was item 32: “To have my wishes respected if I don’t want help”. This makes sense, as the current sample was recruited to a treatment study based on needing rehabilitation, thus probably having little current experience with being exposed to too much help.

The Norwegian version of the FNQ-P was established based on a systematic method of translation/backtranslation, which may have contributed to the overall high construct validity. Regarding construct validity, the findings clearly indicated that the FNQ-P scores were not simply a proxy for aspects of child, parent, or family functioning. Also, the fact that FNQ-P scores did not co-vary with abstract reasoning abilities, executive functioning, or brain injury symptoms in the child indicated that the FNQ-P did not reflect injury severity. A lack of association with overall family functioning also indicates that FNQ-P measures a distinct construct. Of interest, however, is the fact that there was a weak association with emotional functioning, i.e., parental anxiety and parent-rated emotional functioning in the child. This might indicate that the emotional consequences of pABI are not adequately met by the healthcare system. Previous publications from the Oslo region have indicated that, 6 months after TBI, nearly half of the children have unmet needs (37), and that 25% still have unmet needs 2 years after the injury (2). In accordance with the current paper, patients with unmet needs reported more problems with emotional and psychosocial functioning than those without. The work by Dahl et al. (2) was performed before the FNQ-P was translated into Norwegian, thus the findings relate to custom-made operationalizations of needs, and the degree to which these had been met. With a standardized measure in place, future Norwegian studies will be able to describe healthcare needs in the paediatric population in a more standardized manner, and findings will be easier to communicate to the international audience.

### Sample

Of the 111 parents who completed FNQ-P at T1, 77 (69.37 %) completed T2, making the mean ratio of the number of items to sample size 1:1,925. This is considered an acceptable sample size to explore test–retest reliability, also compared with the first psychometric study of FNQ-P by Gan et al. (13). As the sample is based on families participating in a rehabilitation study, families with substance/alcohol abuse, domestic violence issues, and high burden of mental health difficulties, as well as families with a child that does not attend school regularly, were not included. Families with these difficulties were not included in Gan’s study either and should be included in future studies. Likewise, a substantial part of the sample had a high educational level, making conclusions concerning parents with low levels of education challenging. Participants were in the chronic phase of injury, with an average of 5.3 years post injury. In Gan et al. (13), time since injury was 3.1 years, which leaves data comparable, but knowledge about psychometric properties in the acute and post-acute phases is lacking so far.

### Use of FNQ-P

The FNQ-P can also be used in populations other than pABI. The Norwegian version of the FNQ-P has in fact been included in 1 other study examining unmet healthcare needs 6 months after injury in a mixed sample of paediatric and adult patients with moderate to severe physical trauma (38). A recent publication from this study used FNQ-P data from a paediatric sub-sample (*n* = 63) (39), and found that there was a high proportion of needs in this group, particularly related to health information, involvement with care, and emotional support. They also found that a high number of injuries and low age was associated with more needs. There is, however, a challenge in interpreting the data, as any score between 1 and 5 was considered to indicate a need. It is inherently difficult to know how to interpret a score of 5 = completely met. This may indicate that there is in fact a need that has been met, but also that there is no need. In the cognitive interviews that were conducted when establishing the Norwegian translation of FNQ-P, several informants commented that it was difficult to interpret the way time is framed in the questions (15). Respondents are asked to reply related to *current* needs, but informants noted that this was difficult to disentangle from needs that were present at an earlier stage. One way to help respondents with this challenge can be to administer FNQ-P as a clinical interview. However, the high reliability scores found in the current study nonetheless suggest that the questions were interpreted rather consistently across the 2 measurement points. Irrespective of reliability, it should be noted that mean scores on the FNQ-P should be interpreted with caution, and that it might be worth exploring mean scores based on calculations where scores of 0 = not applicable and 5 = fully met are excluded when interpreting unmet needs.

### Limitations

Thirty-four parents did not respond to the retest condition at T2, which left the sample smaller than its full potential in test–retest analysis, but still within acceptable limits. Furthermore, the sample is characterized by being in the chronic phase of injury, and parents were overall highly educated, indicating high socioeconomic status. The study was conducted in an urban part of Norway, and the sample was culturally homogeneous. Also, the validation study was conducted within the context of an RCT including families with ongoing needs, and exclusion criteria will have caused families with the most severely injured children and with severe psychiatric comorbidities to be excluded. Caution is therefore called for regarding generalizability. However, reliability and validity are comparable to existing studies, indicating relevance of findings.

### Conclusion

This study indicates high stability in scores, and that the Norwegian version of the FNQ-P is well suited to measure healthcare needs in the Norwegian paediatric ABI population. There is a general lack of assessment measures with well-documented psychometric properties in the pABI population, and even more so in non-English-speaking countries. The results indicate that FNQ-P can confidently be used to gather reliable and valid data both in the clinical and the scientific setting, and that the properties of the FNQ-P are comparable across countries.
